# A case report of streptococcal toxic shock syndrome caused by *Streptococcus mitis* in a healthy adult

**DOI:** 10.1186/s12879-021-05852-y

**Published:** 2021-02-06

**Authors:** Xiang Chen, Ying Ying Gong, Li Zhang

**Affiliations:** 1 Clinical Medical college, Jining Medical University, N133 Hehua Road, Taibaihu New District, Jining, 272067 Shandong Province China; 2 Department of Gynecology, Affiliated Hospital of Jining Medical University, N89 Guhuai Road, Jining, 272029 Shandong Province China

**Keywords:** *Streptococcus mitis*, Streptococcal toxic shock syndrome, Septic shock, Cellulitis

## Abstract

**Background:**

Streptococcal toxic shock syndrome (STSS) is an acute, multisystem and toxin-mediated disease that usually causes shock and multiple organ failure in the early stages of its clinical course. It is associated with a substantial increase in mortality rate. The disease has been associated with invasive group A Streptococcus and is rarely caused by *Streptococcus mitis* (*S. mitis*). In healthy adults, *S. mitis* is closely related to endocarditis but rarely related to STSS.

**Case presentation:**

We report a case of STSS caused by *S. mitis* in a healthy 45-year-old woman. She presented with fever 14 h after surgery and with hypotension 24 h later, and she subsequently suffered from septic shock, low albumin, dysfunction of coagulation, acute kidney dysfunction, respiratory alkalosis and metabolic acidosis, acute respiratory distress syndrome and cellulitis of the incision. The diagnosis was obtained through clinical manifestation and blood culture examination. The patient was treated with aggressive fluid resuscitation, adequate antibiotics for a total of 4 weeks, respiratory support, and surgical debridement and drainage of the incision. She was discharged after her vital signs returned to normal and the incision healed on day 40 after surgery.

**Conclusions:**

The diagnosis of STSS is often delayed or missed, which leads to a high mortality rate. It is possible to cure patients if the disease can be identified early and treated with aggressive fluid resuscitation, adequate antibiotics and control of the source of infection. Clinicians should consider the disease in the differential diagnosis of septic shock to prevent death.

## Background

Gram-positive infections are responsible for approximately 50% of sepsis cases in the USA [1]. *Streptococcus* species are gram-positive cocci. Toxic shock-like syndrome (TSLS) is the most serious complication of *Streptococcus* infection. Streptococcal toxic shock syndrome (STSS) is a multisystem and severe life-threatening disease [2]. The beginning of the disease is acute, and the progression is rapid and usually presents with shock and multiple organ failure in the early clinical course. Studies have shown that the morbidity rate and mortality rate of STSS are higher than those of TSLS caused by *Staphylococcus* species [3]; the mortality even rate exceeds 25% in the first 24 h [4]. STSS is frequently associated with group A Streptococcus and is rarely caused by *Streptococcus mitis* (*S. mitis*). *S. mitis* belongs to the viridans group of streptococci, part of the normal microbiota of the upper respiratory tract, oral cavity, intestines, skin and female reproductive tract, and is an opportunistic pathogen*. S. mitis* is generally considered to have low pathogenic potential in immunocompetent individuals. Nevertheless, in certain patient populations, it can cause invasive diseases, such as endocarditis and shock. *S. mitis* usually endangers patients who have tumors, organ transplants, immunodeficiency or neutropenia [5–7] but is rare in healthy adults. The case presented herein represents a case of the treatment of a healthy individual with STSS due to *S. mitis*.

## Case presentation

**Fig. 1:**
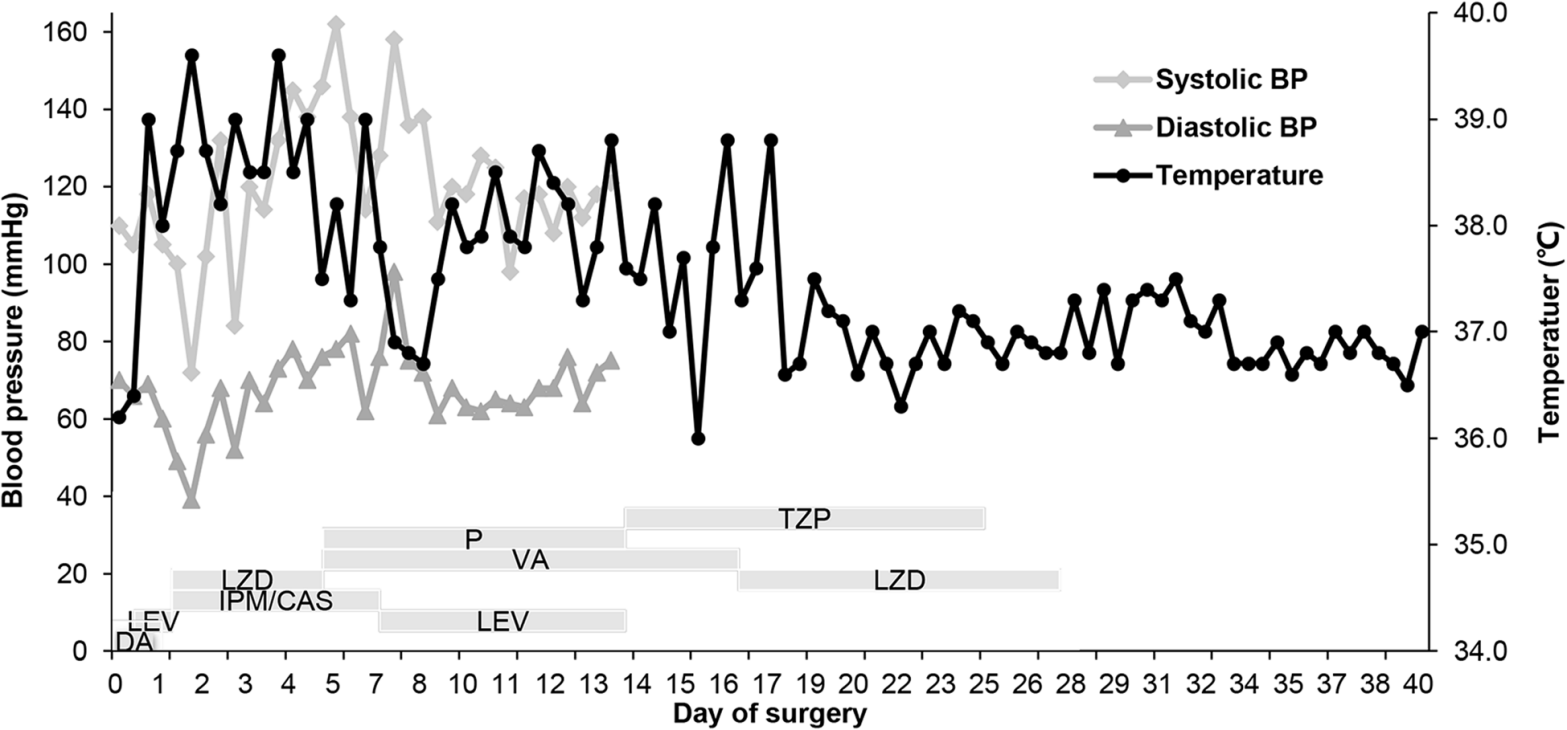
The chart showing temperature, systolic and diastolic pressures after surgery. Grey boxes indicate the usage of antibiotics (Clindamycin (DA), Levofloxacin (LEV), Imipenem/Cilastatin (IMP/CAS), Linezolid (LZD), Vancomycin (VA), Penicillin (P), Piperacillin/Tazobactam (TZP))

A previously healthy 45-year-old woman had an cesarean section delivery. She underwent total abdominal hysterectomy, bilateral salpingectomy and adhesiolysis 3 days after admission because conservative treatment of adenomyosis was ineffective. The patient had a positive cephalosporin skin test and was given clindamycin to prevent postoperative infection. However, she developed a sudden onset chills, a high fever (39.0 °C), and a fast heart rate (115 bpm) but had normal blood pressure (118/69 mmHg) 14 h after the surgery. Laboratory blood samples obtained when the patient was febrile showed that the patient’s white blood cell (WBC) count was 8.0 × 10^9/l with 94.4% neutrophils, and the serum C-reactive protein (CRP) level was 7.0 mg/l. Levofloxacin was added to expand the antibacterial spectrum. However, these symptoms did not improve, and she subsequently developed nausea, vomiting, abdominal distension, abdominal pain, diarrhea and oliguria. At that time, the serum laboratory tests showed that the WBC count (1.9 × 10^9/l) fell below the normal range, and CRP (152 mg/l) was further elevated. Twenty-four hours later, the patient showed anuria. Physical examination detected tachycardia (152 bpm) and hypotension (72/39 mmHg). The patient’s hemodynamic parameters, fever chart and antibiotics administered are shown in Fig. 1.

**Fig. 2:**
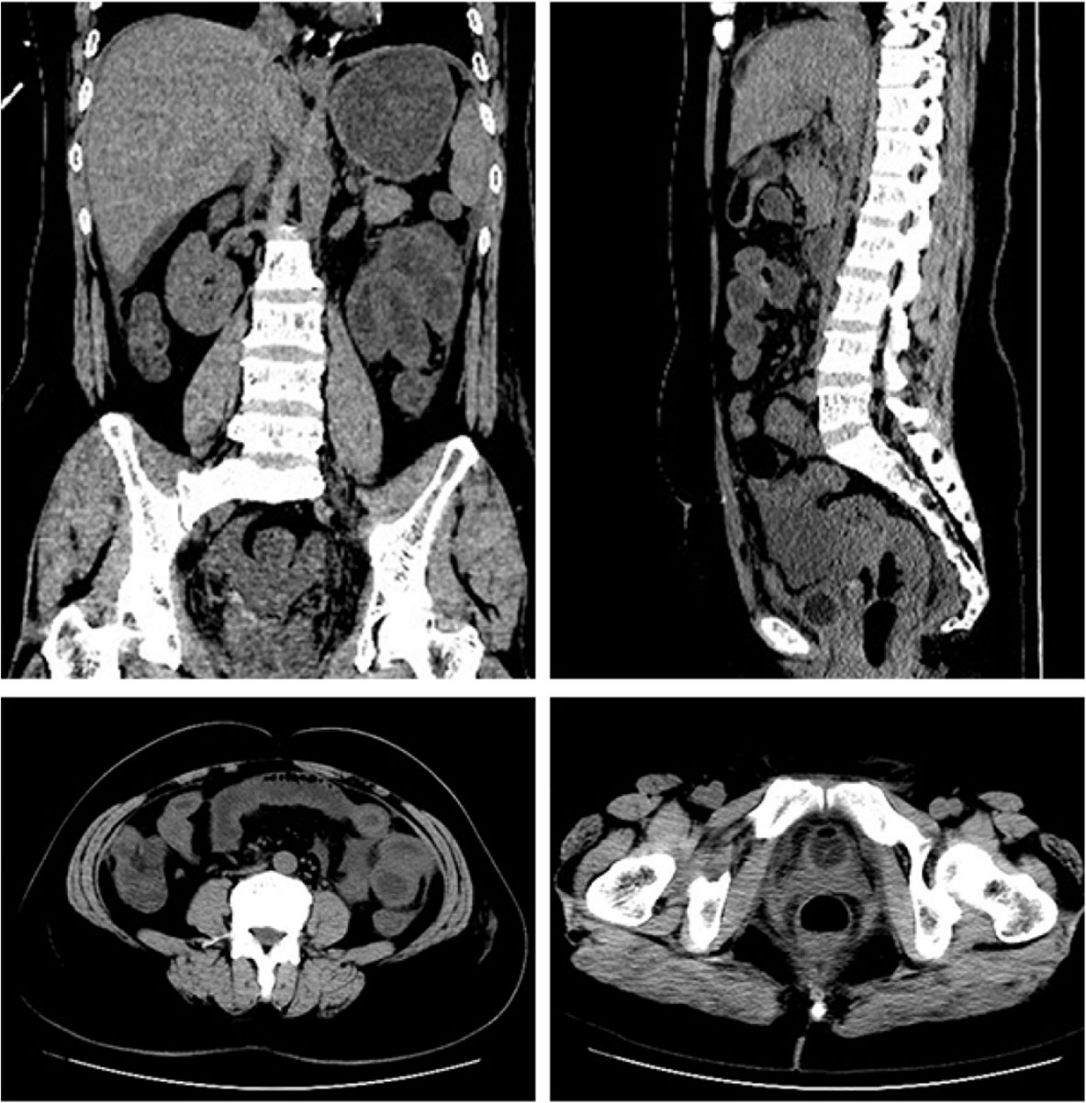
CT plain scan of the pelvic and abdominal cavity

The patient was rapidly admitted to the intensive care unit (ICU). Arterial blood gas indicated high anion gap metabolic acidosis with respiratory alkalosis with a pH of 7.33, anion gap of 16.8 and lactic acid of 4.4. Her laboratory tests showed low albumin (22.3 g/l), high serum creatinine (251.9 μmol/l), dysfunction of coagulation (INR1.97, PT22.4 s), and markedly elevated D-dimer (13.477 mg/l). Computed tomography (CT) of the abdomen and pelvis was performed to rule out the presence of a possible occult abscess (Fig. 2). She was instantly placed empirically on imipenem/cilastatin and linezolid, given fluid resuscitation and started on noradrenaline via a peripheral intravenous catheter. Afterwards, she received intravenous infusion of albumin (80 g) and virus inactivated plasma (300 ml). Three days after surgery, two sets of blood cultures were positive for *S. mitis* in the aerobic and anaerobic bottles with a time to positivity of less than 72 h. Antimicrobial susceptibility testing revealed resistance towards clindamycin, moderate resistance towards erythromycin and sensitivity towards penicillin, levofloxacin, vancomycin and linezolid. Henceforward, the patient was diagnosed with STSS caused by *S. mitis*. Frequent replacement of antibiotics may lead to the emergence of antibiotic-resistant bacteria, so we did not use penicillin immediately.

On day 4 after surgery, the patient developed moderate acute respiratory distress syndrome (ARDS) with a PaO2/FiO2 ratio of 162.5 mmHg. Pulmonary edema and bilateral pleural effusions were observed on chest radiographs. It was necessary to intubate and ventilate the patient. As the PaO2/FiO2 ratio increased to 377 mmHg and pulmonary edema and pleural effusions were improved on day 8 after surgery, ventilation could be stopped.

Due to new-onset thrombocytopenia (48 × 10^9/l) thought to be associated with STSS and continued fever despite receiving adequate antibiotic therapy, linezolid was discontinued, and imipenem/cilastatin, vancomycin and penicillin were administered on day 5 after surgery. Two days later, her temperature dropped to the normal range. Additionally, there was no evidence of gram-negative bacilli infection, so we replaced imipenem/cilastatin with levofloxacin based on antimicrobial susceptibility testing. However, her temperature rose again 1 day later (Fig. 1). Soon afterwards, her temperature fluctuated between low and medium heat. On day 13 after surgical intervention, physical examination showed that she developed skin redness and subcutaneous induration on the upper left side of the incision. We considered it to be cellulitis of the incision according to ultrasound imaging and the clinical manifestation, for which she underwent debridement. In addition, we used piperacillin/tazobactam instead of penicillin and levofloxacin. The hemolytic streptococcal spread of infection to the incision cannot be excluded, although the local secretion smear and cultures were sterile. Her temperature gradually dropped, and her blood pressure and lactic acidosis recovered to the normal range; hence, she was transferred to the general ward on the 15th postoperative day.

One day later, her temperature suddenly rose to 38.8 °C again despite adequate drainage of the incision (Fig. 1). We considered that the reason for the fever was still related to cellulitis of the incision. Therefore, linezolid was used in place of vancomycin to increase the drug concentration in the skin and soft tissues. Her temperature no longer rose to 38 °C 18 days after surgery (Fig. 1). The antibiotics were stopped on the 27th day after surgery. In summary, the patient was treated with antibiotics for a total of 4 weeks. She was discharged when her vital signs were stable, and the incision healed on day 40 after surgery.

## Discussion and conclusions

STSS commonly occurs after viral infections (e.g., varicella, influenza), pharyngitis, and local soft tissue trauma. STSS is associated with deeper sites of infection (e.g., infection after penetrating injuries, necrotizing fasciitis) [8]. However, 50% of clinical cases of STSS have no clear primary infection focus [9]. The exact mechanism of STSS is not fully understood. At present, it is believed that STSS is related to the ability of streptococcal toxins to be superantigens, the complex interaction between host immunity and pathogen virulence, and the host’s response to streptococcal infection [2, 10].

TSLS with *S. mitis* has been documented in bone marrow transplantation, neutropenic, cancer and chemotherapy patients [6, 7, 11, 12]. However, the occurrence of TSLS with *S. mitis* in previously healthy adults is rare. Madhusudhan et al. [13] described a previously healthy 33-year-old woman with a history of 2 days of feeling lethargic and unwell, followed by vomiting, diarrhea, shortness of breath, swelling of the face, necrotizing fasciitis and hypotension. Finally, the patient was successfully treated with imipenem/cilastatin and clindamycin, aggressive intensive care support, renal replacement therapy by veno-venous filtration, and so on. In China, there was an outbreak of TSLS with *S. mitis* in previously healthy adults in 1990 [13]. In this case, approximately half of the patients presented with symptoms or signs of streptococcal TSLS characterized by hypotension and multiorgan failure. However, there were very few deaths.

The diagnosis of STSS is mainly based on clinical presentation and blood culture. However, it has no specific clinical features, which often causes a delay in diagnosis. The clinical diagnostic criteria for STSS revised by the Centers for Disease Control and Prevention (CDC) in 2010 have been widely accepted [14].

International guidelines for the management of sepsis and septic shock were updated in 2016 [15]. The rapid progression from onset to multiorgan failure in STSS necessitates immediate action with treatment and fluid resuscitation [8, 15–17]. Active use of adequate antibiotics and control of the source of infection are the basis for treating the disease [17]. Tracheal intubation or renal replacement therapy may be performed, if necessary. Intravenous antimicrobials should be administered as soon as possible within 1 h after recognition. Before the bacteria are identified and tested for drug resistance, empirical broad-spectrum therapy with one or more antimicrobials (e.g., piperacillin/tazobactam, vancomycin, and anidulafungin) for patients presenting with sepsis and septic shock to cover all likely pathogens is recommended [15]. A broad consensus has been reached about the use of combination therapy including inhibition of bacterial toxin production (e.g., clindamycin with β-lactams) for STSS [15].

Statistical data from the antibacterial resistance investigation collected by the China Antimicrobial Surveillance Network (CHINET), which involved 44 hospitals, showed that there was low resistance of each Streptococcus group to penicillin but high resistance (all above 56%) to erythromycin and clindamycin in 2018. Penicillin is still the first-line antibiotic of choice for STSS [16, 17]. Although the resistance rate of Streptococcus to clindamycin is high, clindamycin still plays a significant role in STSS management. Clindamycin has been shown to suppress superantigen production and possesses better tissue penetration and longer postantibiotic effects than penicillin [18]. It can also reduce the production of superantigens even in drug-resistant bacteria [19]. The combination of clindamycin and penicillin can achieve a rapid bactericidal effect, prevent the further production of superantigens by inhibiting the synthesis of bacterial proteins [16], improve the prognosis of the disease and reduce the mortality rate [20]. For methicillin-resistant *Staphylococcus aureus* (MRSA), it is recommended to use glycopeptides (such as vancomycin) instead of β-lactams [16, 17]. Immunoglobulin can neutralize toxins and inhibit the superantigen response, but the efficacy of intravenous immunoglobulin (IVIG) in STSS has not yet been determined [18]. The use of IVIG in STSS cannot be routinely recommended and should be discussed on a case-by-case basis [21].

For patients with deep soft tissue infections, surgical debridement should be performed immediately.

This case did not show obvious primary infection focus combined with history. Since *S. mitis* is part of the normal microbiota of the skin and the patient developed cellulitis of the incision later in the course of the disease, we speculate that the source of infection may be *S. mitis* infection at the operation site.

According to the STSS diagnostic criteria revised by the CDC in 2010, the patient’s diagnosis of STSS is clear. In addition, we found that the patient did not have a characteristic rash, and her clinical manifestations were consistent with the clinical features of STSS caused by *S. mitis* that appeared in China in 1990 [13].

The clinical practice guidelines for the prevention of infection after gynecologic procedures, developed by the American College of Obstetricians and Gynecologists (ACOG), recommend a single dose of cefazolin as antibiotic prophylaxis for abdominal hysterectomy surgeries [22]. Clindamycin or metronidazole alone have been shown to reduce infection after hysterectomy, but broader spectrum coverage results in even lower infection rates. Therefore, the combination of metronidazole or clindamycin plus gentamicin or aztreonam is recommended for patients in whom cephalosporins are contraindicated [22]. Our patient with a positive cephalosporin skin test received clindamycin alone to prevent infection after surgery. In addition, an antimicrobial susceptibility test revealed that *S. mitis* was resistant to clindamycin. These factors were considered the main reasons why our patient suffered from STSS. STSS will lead to her death if not diagnosed early and effectively treated in time. In addition, we did not find cellulitis earlier, so the patient had a recurrent fever. When a patient’s temperature rises again after returning to normal, clinicians should suspect that the rise in temperature is associated with incisional infection. Although we immediately performed local debridement and drainage to keep the wound from worsening when we found cellulitis in the surgical incision, there are shortcomings to our treatment. We did not continue to use clindamycin in subsequent treatments despite it possibly having some effect in eradicating clindamycin-resistant bacteria.

An update to the Infectious Diseases Society of America guidelines for the diagnosis and management of skin and soft tissue infections was published in 2014 [23]. Penicillin or clindamycin is recommended for the treatment of nonpurulent skin infection caused by Streptococcus, and vancomycin plus either piperacillin/tazobactam or imipenem/meropenem is recommended as a reasonable empiric regimen for severe cellulitis [23]. On postoperative day 13, incisional cellulitis was discovered with continued fever in our patient, so we changed antibiotics according to cellulitis treatment guidelines in addition to draining the incision. However, the temperature rose from normal to 38.8 °C again after 3 days. Then, we replaced vancomycin with linezolid because studies showed that linezolid has better penetration into skin and soft tissue than vancomycin [24, 25]. The therapeutic effect of linezolid on the patient was remarkable.

In summary, STSS caused by *S. mitis* is a rare disease and is associated with significant mortality. It can cause multiple organ failure in a short time frame, and we need to be alert to the possibility of this disease in a healthy adult. Due to nonspecific clinical features, unclear sources of infection, lack of early skin lesions, and the time required for laboratory culture often leads to delays in the diagnosis of STSS, clinicians need to remain vigilant. The prognosis of the disease is poor. Early identification of the disease, instantaneous start of effective antibiotic treatment and rapid control of the source of infection are the keys to reducing the morbidity and mortality of this deadly disease.

## Data Availability

Data sharing is not applicable to this article as no datasets were generated or analyzed during the current study.

## Abbreviations

TSLS: Toxic shock-like syndrome
STSS: Streptococcal toxic shock syndrome
*S. mitis*: *Streptococcus mitis*
MRSA: Methicillin-resistant *Staphylococcus aureus*
CRP: C-reactive protein
WBC: White blood cell
ICU: Intensive care unit
INR: International normalized ratio
PT: Prothrombin time
CT: Computed tomography
ARDS: Acute respiratory distress syndrome
CDC: Centers for Disease Control and Prevention
ACOG: American College of Obstetricians and Gynecologists
CHINET: China Antimicrobial Surveillance Network
IVIG: Intravenous immunoglobulin

## References

[1] Martin GS, Mannino DM, Eaton S, Moss M (2003). The epidemiology of sepsis in the United States from 1979 through 2000. N Engl J Med.

[2] Low DE (2013). Toxic shock syndrome: major advances in pathogenesis, but not treatment. Crit Care Clin.

[3] Chuang Y, Huang Y, Lin T (2005). Toxic shock syndrome in children: epidemiology, pathogenesis, and management. Paediatric Drugs.

[4] Lamagni TL, Neal S, Keshishian C, Powell D, Potz N, Pebody R (2009). Predictors of death after severe Streptococcus pyogenes infection. Emerg Infect Dis.

[5] Freifeld AG, Razonable RR (2014). Viridans group streptococci in febrile neutropenic cancer patients: what should we fear?. Clin Infect Dis.

[6] Guerrero-Del-Cueto F, Ibanes-Gutierrez C, Velazquez-Acosta C, Cornejo-Juarez P, Vilar-Compte D (2018). Microbiology and clinical characteristics of viridans group streptococci in patients with cancer. Braz J Infect Dis.

[7] Martino R, Manteiga R, Sánchez I, Brunet S, Sureda A, Badell I (1995). Viridans streptococcal shock syndrome during bone marrow transplantation. Acta Haematol.

[8] Gottlieb M, Long B, Koyfman A (2018). The evaluation and management of toxic shock syndrome in the emergency department: a review of the literature. J Emerg Med.

[9] Lamagni TL, Darenberg J, Luca-Harari B, Siljander T, Efstratiou A, Henriques-Normark B (2008). Epidemiology of severe Streptococcus pyogenes disease in Europe. J Clin Microbiol.

[10] Commons RJ, Smeesters PR, Proft T, Fraser JD, Robins-Browne R, Curtis N (2014). Streptococcal superantigens: categorization and clinical associations. Trends Mol Med.

[11] Petrosyan V, Holder M, Ajami NJ, Petrosino JF, Sahasrabhojane P, Thompson EJ, et al. Complete genome sequence of *Streptococcus mitis* strain SVGS_061 Isolated from a neutropenic patient with viridans group streptococcal shock syndrome. Genome Announc. 2016;4(2). 10.1128/genomeA.00259-16. 10.1128/genomeA.00259-16 PMC4824267 27056234

[12] Nielsen MJ, Claxton S, Pizer B, Lane S, Cooke RP, Paulus S (2016). Viridans group streptococcal infections in children after chemotherapy or stem cell transplantation: a 10-year review from a tertiary pediatric hospital. Medicine (Baltimore).

[13] Lu HZ, Weng XH, Zhu B, Li H, Yin YK, Zhang YX (2003). Major outbreak of toxic shock-like syndrome caused by *Streptococcus mitis*. J Clin Microbiol.

[14] Streptococcal Toxic Shock Syndrome (STSS) (*Streptococcus pyogenes*) 2010 Case Definition. https://wwwn.cdc.gov/nndss /conditions /streptococcal-toxic-shock-syndrome/case-definition /2010/.

[15] Rhodes A, Evans LE, Alhazzani W, Levy MM, Antonelli M, Ferrer R (2017). Surviving sepsis campaign: international guidelines for management of sepsis and septic shock: 2016. Intensive Care Med.

[16] Lappin E, Ferguson AJ (2009). Gram-positive toxic shock syndromes. Lancet Infect Dis.

[17] Wilkins AL, Steer AC, Smeesters PR, Curtis N (2017). Toxic shock syndrome – the seven Rs of management and treatment. J Infect.

[18] Note S, Soentjens P, Van Laer M, Meert P, Vanbrabant P (2019). Streptococcal toxic shock syndrome in a returning traveller. Acta Clin Belg.

[19] Andreoni F, Zürcher C, Tarnutzer A, Schilcher K, Neff A, Keller N (2017). Clindamycin affects group a streptococcus virulence factors and improves clinical outcome. J Infect Dis.

[20] Carapetis JR, Jacoby P, Carville K, Ang SJ, Curtis N, Andrews R (2014). Effectiveness of clindamycin and intravenous immunoglobulin, and risk of disease in contacts, in invasive group a streptococcal infections. Clin Infect Dis.

[21] Schmitz M, Roux X, Huttner B, Pugin J (2018). Streptococcal toxic shock syndrome in the intensive care unit. Ann Intensive Care.

[22] Practice Bulletin No ACOG (2018). 195: prevention of infection after gynecologic procedures. Obstet Gynecol.

[23] Stevens DL, Bisno AL, Chambers HF, Dellinger EP, Goldstein EJ, Gorbach SL (2014). Practice guidelines for the diagnosis and management of skin and soft tissue infections: 2014 update by the Infectious Diseases Society of America. Clin Infect Dis.

[24] MacGowan AP (2003). Pharmacokinetic and pharmacodynamic profile of linezolid in healthy volunteers and patients with Gram-positive infections. J Antimicrob Chemother.

[25] Stein GE, Schooley SL, Peloquin CA, Kak V, Havlichek DH, Citron DM (2005). Pharmacokinetics and pharmacodynamics of linezolid in obese patients with cellulitis. Ann Pharmacother.

